# Use of Electronic Health and Its Impact on Doctor-Visiting Decisions Among People With Diabetes: Cross-Sectional Study

**DOI:** 10.2196/13678

**Published:** 2019-04-26

**Authors:** Anne Helen Hansen, Tor Claudi, Eirik Årsand

**Affiliations:** 1 Centre for Quality Improvement and Development University Hospital of North Norway Tromsø Norway; 2 Department of Community Medicine Faculty of Health Sciences UiT–The Arctic University of Norway Tromsø Norway; 3 Department of Medicine Nordland Hospital Bodø Norway; 4 Norwegian Centre for E-health Research University Hospital of North Norway Tromsø Norway; 5 Department of Clinical Medicine UiT–The Arctic University of Norway Tromsø Norway

**Keywords:** eHealth, internet, internet information, doctor-seeking behavior, cross-sectional study, diabetes, Norway

## Abstract

**Background:**

Despite the increasing prevalence of diabetes and increasing use of electronic health (eHealth) among people with diabetes, little is known about the association between the use of eHealth and the use of provider-based health services.

**Objective:**

The objective of this study was to investigate whether the use of eHealth might change patients’ decisions regarding doctor-seeking behavior and whether information acquired from the internet was discussed with a doctor.

**Methods:**

We used email survey data collected in 2018 from members of the Norwegian Diabetes Association (aged 18 to 89 years) diagnosed with diabetes. Using logistic regressions, we studied patients’ internet-triggered changes in decisions regarding doctor visits; whether they discussed information from the internet with a doctor; and whether these topics were associated with gender, age, education, self-rated health, and self-reported anxiety/depression.

**Results:**

Among the 895 informants, 75.4% (645/856) had never made an internet-triggered change of decision in any direction regarding visiting a doctor, whereas 16.4% (41/859) had decided to visit and 17.3% (148/856) had decided not to visit. The probability of changing decisions decreased with higher age and increased with the severity of self-reported anxiety/depression. Around half of the study participants (448/858, 52.2%) had never discussed information from the internet with a doctor. The probability of discussing internet information with a doctor was higher for those in bad/very bad self-rated health (odds ratio 2.12, CI 1.15-3.90) and for those with moderate self-reported anxiety/depression (odds ratio 2.30, CI 1.30-4.10).

**Conclusions:**

Our findings suggest that using eHealth has a significant impact on doctor-visiting decisions among people with diabetes, especially among people aged 18 to 39 years and among those reporting anxiety/depression. It is of great importance that the information posted is of high quality and that the large differences between internet-users regarding age as well as mental and somatic health status are taken into account. More research is needed to confirm and further explore the findings of this study.

## Introduction

### Increasing Use of Electronic Health

The use of eHealth has developed and increased rapidly over the past decades. The term eHealth refers to “the transfer of health resources and health care by electronic means,” and internet use for health information is a part of this [1]. In this paper, we consider eHealth in the form of apps, search engines, social media, and video services. Of particular interest are patients with chronic disease, such as diabetes, who are in particular need of health information, monitoring, and follow-up of health and disease parameters.

Around 80% of the general population in the United States and Europe conduct health-related searches [2-5]. It was recently reported that 87% of Norwegians with type 1 diabetes (T1D) used eHealth in one or more forms, and 84% had used search engines (such as Google) sometimes or often during the previous year [6]. Most Norwegian households (98%) have internet access [7], 96% of the population aged 16 to 79 years have used the internet during the previous 3 months, and 90% use the internet every day [8].

### Increasing Prevalence of Diabetes

The prevalence of diabetes is increasing worldwide and expected to rise to 642 million cases in 2040 [9]. Global prevalence in adults is estimated at 8.8% [9]. Around 245,000 persons have been diagnosed with diabetes in Norway, of whom around 28,000 have T1D [10]. Despite a decreasing trend in the incidence of type 2 diabetes (T2D) in Norway, the prevalence increased from 4.9% to 6.1% from 2009 to 2014 [11]. Most patients do not reach the combined national treatment targets for prevention of complications [12-14].

### Relationship Between the Use of eHealth and Doctor-Visiting Behavior

A positive association between the use of eHealth and general practitioner (GP) visits has been reported [15]. Others have found an inverse association with the frequency of doctor visits [16,17]. eHealth might be used to decide about the need for seeing a doctor, seek information before the visit, or seek reassurance or additional information after the visit [6,18].

Some have suggested that the use of eHealth may postpone or replace medical consultations [19]. A French study found that around 30% of young eHealth users (aged 15 to 30 years) often used the internet instead of visiting a doctor [20]. In the same study, 88.6% reported that eHealth use did not change their consultation frequency, whereas 4.9% reported seeing a doctor more often and 6.5% less often. A German study found that frequent users of health services were 73% more likely to seek health information on the internet compared to nonusers [21]. This is in line with the illness behavior model [22], where people in poor health are more likely to seek disease-related information online and use health services more frequently. In a previous study among people with T1D, we found that the use of search engines was associated with more specialist visits, whereas GP visits were not associated with the use of eHealth [6]. Whether use of the internet for health information in a Norwegian population with diabetes might lead to or prevent doctor visits is unknown.

The use of eHealth often takes place without doctor involvement. Research from the United States reports that only 31% of mobile health (mHealth, which refers to mobile and wireless communication technologies to aid in health and health care) users prioritized their physician’s involvement [23]. To what extent diabetes patients in Norway discuss internet information with their doctor is unknown.

Due to the increased prevalence of diabetes and increased use of eHealth, doctor-visiting behavior related to the use of eHealth among people with chronic diseases such as diabetes is highly relevant for research. The use of eHealth among people with diabetes might have an impact on health information level, handling and care of health and disease, and doctor-seeking behavior and thus affects the overall important outcomes morbidity and mortality.

### Aim

The aim of this study was to investigate whether the use of eHealth might lead to or prevent doctor visits and whether such changes in doctor-seeking behavior might be associated with gender, age, education, self-rated health, and self-reported anxiety/depression among people with diabetes. Furthermore, we aimed to investigate whether information from the internet was discussed in the clinical encounter and whether this was associated with gender, age, education, self-rated health, and self-reported anxiety/depression.

## Methods

### Data

This cross-sectional study is part of the DIAcare project [24], which uses data obtained in 2018 from members of the Norwegian Diabetes Association (NDA). As of December 31, 2017, the organization had 33,908 members, of whom about 30% have T1D [25]. The Norwegian Centre for Research Data (NSD) distributed the invitations to a randomly selected sample of 5971 individuals with email addresses registered with NDA, who answered through NSD’s secure Web survey system. As described in our protocol paper, we planned to use data from the seventh Tromsø Study, conducted in 2015-2016 [26]. However, the Tromsø Study could not give us access, and we decided to develop a tailored questionnaire based on the specific objectives of our study using relevant questions from other published surveys [26,27].

Information about the study was posted together with the invitation. The questionnaire (Multimedia Appendix 1) included questions about health status, disease duration, severity and treatment of diabetes, use of and experiences with eHealth and health care services, and demographic and socioeconomic information. We reviewed and tested the questionnaire several times before distribution to the informants. Nonrespondents were given one reminder, sent by email 15 days after the first request.

### Participants

**Figure 1:**
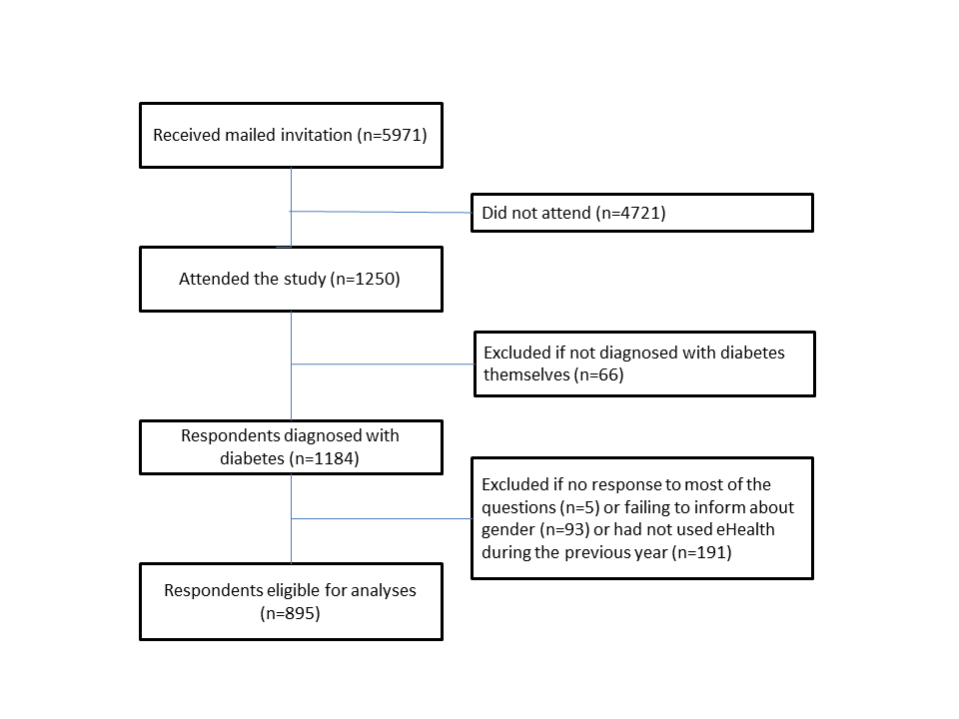
Flowchart of study population.

The respondents could not fill in the questionnaire more than once. Starting from 1250 participants, we excluded the 66 individuals who had not been diagnosed with diabetes themselves (family members, health personnel, and others). We also excluded those who left out most of the questions (n=5) and those who did not give information about gender (n=93). Finally, we excluded those who had not used eHealth in the form of apps, search engines, social media, or video services “sometimes or often” during the previous year (n=191). The analyzed sample consisted of 895 respondents with diabetes (Figure 1).

### Variables

We used 3 dependent variables developed from the following questions: Based on the information you have found on the internet, have you (1) decided to visit a doctor, when you would otherwise not have visited one, (2) decided not to visit a doctor, when you would otherwise have visited one, or (3) discussed the information with a doctor?

We dichotomized these variables by merging the original 4 response options into “never” and “once, sometimes or often” for two reasons. First, we were more interested in investigating if the participants had ever changed their doctor-seeking behavior or discussed internet information with a doctor than finding out how often they had done so. Second, merging the answering options into 2 groups gave an easier and more reader-friendly interpretation of the regression results.

Age was grouped in 20-year intervals. The 4 education categories were labeled low (primary/part of secondary school), middle (high school), high (college/university less than 4 years), and highest (college/university 4 years or more). Response options for self-rated health were excellent, good, fair, bad, and very bad. Due to low numbers in the very bad category (4 respondents), we merged the bad and very bad categories. Response options for self-reported degree of anxiety/depression were none, slight, moderate, severe, and extreme. We merged the severe and extreme categories due to few (4) respondents in the extreme category.

### Analyses

We analyzed data by means of descriptive statistics and logistic regressions and tested correlations with the Spearman correlation test.

We constructed 3 regression models, with the 3 different dependent variables: “Decided to visit a doctor,” “Decided not to visit a doctor,” and “Discussed internet information with a doctor.” The independent variables were gender, age, education, self-rated health, and self-reported degree of anxiety/depression. All the independent variables were introduced collectively into the multivariable models.

We subsequently introduced the response time variable into the regression models, comparing the late respondents with the early respondents, assuming that those who did not respond at first were more similar to nonrespondents [28].

We used 95% confidence intervals and set *P*<.05 as the significance level throughout the study. All analyses were accomplished using Stata version 14.2 (StataCorp LLC).

### Ethics

The Regional Committee for Medical and Health Research Ethics found that an application for this project was not required according to the Norwegian Health Research Act (ref 2015/1779/REK nord). The data protection officer at the University Hospital of North-Norway approved the study (ref 2017/6579). The data bureau NSD received no information about the participants other than the email addresses.

## Results

### Participation

A total of 1250 persons aged 18 to 89 years answered the questionnaire, constituting a minimum response rate of 20.93%. We assume the real response rate to be higher since we had more than 400 bounce backs from email servers unable to deliver the invitation, and we do not know how many actually received the survey email. Eligible for analysis were the 895 persons who reported having been diagnosed with diabetes themselves.

### Characteristics of the Participants

Mean age of participants was 53.5 years, 50.4 years for women and 56.3 years for men. Median age was 56 years. Mean disease duration was 13.6 (median 10) years.

The largest groups were made up of men (460/895, 51.4%), persons aged 60 years and over (369/895, 41.2%), persons with high education (265/832, 31.8%), diabetes duration of less than 10 years (295/892, 33.1%), good self-rated health (446/887, 50.3%), and no anxiety/depression (603/882, 68.4%). Most participants (718/859, 83.6%) had never made an internet-triggered decision to visit a doctor when they would otherwise not have visited one, whereas 16.4% (141/859) had made such a decision (Table 1). On the other hand, 82.7% (708/856) had never decided not to visit a doctor when they would otherwise have visited one, meaning that 17.3% (148/859) had made such a decision. When adding these 2 variables, we found that 75.4% (645/856) had never changed their decision regarding visiting a doctor in any direction based on information from the internet. These figures indicate that some had changed decisions in both directions.

A little more than half of the participants had never discussed information from the internet with a doctor (448/858, 52.2%).

### Probability of an Internet-Triggered Change in the Decision to Visit or Not to Visit a Doctor

The probability of an internet-triggered decision to visit a doctor when one would otherwise not have visited one decreased with higher age and increased with the severity of self-reported anxiety/depression. People aged 60 years and over were significantly less likely to change their decision in this direction, compared with people aged 18 to 39 years (odds ratio [OR] 0.39, CI 0.23-0.67). The probability of changing the decision in this direction was more than 3 times higher for those with severe anxiety/depression (OR 3.2, CI 1.28-8.04), compared with no anxiety/depression. Gender, education, and self-rated health were not associated with deciding to visit a doctor based on information from the internet (Table 2).

The probability of an internet-triggered decision in the opposite direction (not to visit when one would otherwise have visited a doctor) followed the same pattern, with the exception that men were less likely to change their decision in this direction, compared with women (OR 0.63, CI 0.43-0.93). People aged 60 years and over were less likely to decide not to visit a doctor when they would otherwise have visited one, compared with people aged 18 to 39 years (OR 0.36, CI 0.22-0.59). Likewise, the probability of changing their decision in this direction was around 3 times higher for people with severe anxiety/depression (OR 2.97, CI 1.18-7.47), compared with no anxiety/depression. Education and self-rated health were not associated with deciding not to visit a doctor based on information from the internet.

### Probability of Discussing Information From the Internet With a Doctor

People with bad/very bad self-rated health had a significantly higher probability of discussing information from the internet with a doctor, compared with those in excellent self-rated health (OR 2.12, CI 1.15-3.90). Discussing internet information with a doctor was associated with a moderate degree of self-reported anxiety/depression, compared with no anxiety/depression (OR 2.30, CI 1.30-4.10). Gender, education, and self-rated health were not associated with discussing information from the internet with a doctor (Table 3).

All findings presented in Tables 2 and 3 persisted after introduction of the response time variable into the regression models. There were no strong correlations (defined as Spearman rho>.5) between the independent variables. The strongest correlations were found for the variables self-rated health and self-rated anxiety/depression (rho=.3442). There were no other correlations above rho=.1471 (age and gender).

**Table 1:** Sample characteristics.

Characteristic			Value, n (%)
**Gender (n=895)**			
	Female		435 (48.6)	
	Male		460 (51.4)	
**Age in years (n=895)**			
	18-39		180 (20.1)	
	40-59		346 (38.7)	
	60 and over		369 (41.2)	
**Education (n=832)^a^**			
	Low		74 (8.9)	
	Middle		241 (29.0)	
	High		265 (31.8)	
	Highest		252 (30.3)	
**Duration of diabetes, years (n=892)**			
	<10		295 (33.1)	
	10-19		253 (28.3)	
	20-29		170 (19.1)	
	30 and over		174 (19.5)	
**Self-rated health (n=887)**			
	Excellent		125 (14.1)	
	Good		446 (50.3)	
	Fair		224 (25.2)	
	Bad/very bad		92 (10.4)	
**Self-reported degree of anxiety/depression (n=882)**			
	None		603 (68.5)	
	Slight		181 (20.5)	
	Moderate		72 (8.2)	
	Severe		26 (2.9)	
**Based on information from the internet, have you decided to visit a doctor? (n=859)**			
	Never		718 (83.6)	
	Once		81 (9.4)	
	Sometimes		58 (6.8)	
	Often		2 (0.2)	
**Based on information from the internet, have you decided not to visit a doctor? (n=856)**			
	Never		708 (82.7)	
	Once		47 (5.5)	
	Sometimes		88 (10.3)	
	Often		13 (1.5)	
**Have you discussed information from the internet with a doctor? (n=858)**			
	Never		448 (52.2)	
	Once		132 (15.4)	
	Sometimes		254 (29.6)	
	Often		24 (2.8)	

^a^Low (primary/part of secondary school), middle (high school), high (college/university less than 4 years), highest (college/university 4 years or more).

**Table 2:** Probability of changing the decision to visit or not to visit a doctor based on information from the internet.

Characteristics		Decided to visit a doctor when you would otherwise not have visited one (n=821)			Decided not to visit a doctor when you would otherwise have visited one (n=819)		
		OR^a^	*P* value	CI	OR	*P* value	CI
**Gender**							
	Female	1.00	—^b^	—	1.00	—	—
	Male	0.75	.15	0.50-1.11	0.63	.02	0.43-0.93
**Age in years**							
	18-39	1.00	—	—	1.00	—	—
	40-59	0.56	.02	0.34-0.92	0.43	.001	0.27-0.70
	60 and over	0.39	.001	0.23-0.67	0.36	<.001	0.22-0.59
**Education^c^**							
	Low	1.00	—	—	1.00	—	—
	Middle	1.11	.79	0.50-2.45	1.05	.90	0.50-2.21
	High	1.94	.09	0.90-4.17	1.10	.79	0.53-2.31
	Highest	1.04	.92	0.47-2.31	1.28	.51	0.61-2.68
**Self-rated health**							
	Excellent	1.00	—	—	1.00	—	—
	Good	1.31	.44	0.66-2.58	1.35	.37	0.70-2.60
	Fair	1.73	.14	0.83-3.59	1.58	.21	0.78-3.22
	Bad/very bad	2.04	.09	0.89-4.66	1.85	.14	0.82-4.17
**Self-reported degree of anxiety/depression**							
	None	1.00	—	—	1.00	—	—
	Slight	1.85	.01	1.15-2.96	2.03	.002	1.29-3.21
	Moderate	2.54	.005	1.33-4.83	2.27	.01	1.19-4.31
	Severe	3.2	.01	1.28-8.04	2.97	.02	1.18-7.47

^a^OR: odds ratio.

^b^Not applicable (reference group).

^c^Low (primary/part of secondary school), middle (high school), high (college/university less than 4 years), highest (college/university 4 years or more).

**Table 3:** Probability of discussing information from the internet with a doctor (n=821).

Characteristic		OR^a^	*P* value	95% CI
**Gender**				
	Female	1.00	—^c^	—
	Male	1.01	.97	0.76-1.34
**Age**				
	18-39	1.00	—	—
	40-59	1.01	.95	0.68-1.51
	60 and over	0.89	.56	0.51-1.32
**Education^b^**				
	Low	1.00	—	—
	Middle	0.8	.41	0.46-1.37
	High	1.39	.23	0.81-2.37
	Highest	1.13	.65	0.66-1.94
**Self-rated health**				
	Excellent	1.00	—	—
	Good	1.21	.38	0.79-1.85
	Fair	1.27	.33	0.79-2.05
	Bad/very bad	2.12	.02	1.15-3.90
**Self-reported degree of anxiety/depression**				
	None	1.00	—	—
	Slight	1.29	.17	0.90-1.85
	Moderate	2.3	.005	1.30-4.10
	Severe	1.66	.25	0.71-3.90

^a^OR: odds ratio.

^b^Low (primary/part of secondary school), middle (high school), high (college/university less than 4 years), highest (college/university 4 years or more).

^c^Not applicable (reference group).

## Discussion

### Principal Findings

Most study participants (645/856, 75.4%) had never changed their decision regarding visiting a doctor based on information from the internet, whereas some had decided to visit (141/859, 16.4%,) and/or not to visit (148/856, 17.3%). The probability of changing the decision decreased with higher age and increased with the severity of self-reported anxiety/depression. Around half of the study participants (448/858, 52.2%) had never discussed information from the internet with a doctor. The probability of discussing internet information with a doctor increased for those in bad/very bad self-rated health (compared with excellent health) and for those with moderate self-reported anxiety/depression (compared with no anxiety/depression).

### Internet-Triggered Changes in Doctor-Visiting Decisions

Approximately 3 out of 4 participants in this study reported that they had never changed their decision to visit or not to visit a doctor based on information from the internet, whereas 16.4% had decided to visit and 17.3% had decided not to visit. It is worth noting that some reported changes in both directions, and that the 2 groups were about the same size (Table 1). A study among elderly internet users in the Netherlands (data collected in 2011) found that 48% had decided to go to the doctor and 24% had decided not to go because of internet information [29]. The percentage of participants who decided to visit a doctor was nearly 3 times higher in the Dutch study compared with our results. On the other hand, a French study among young internet users found that 4.9% visited a doctor more often and 6.5% less often based on information from the internet [20]. Around 90% of participants in studies from the United States, Japan, and France reported that use of the internet for health purposes did not change their health care–seeking behavior (United States 94%, Japan 88.9%, France 88.6%), meaning that 10% actually did [20,30,31].

Direct comparison between these studies is challenging due to methodological and cultural heterogeneity. Our study sampled people with diabetes, whereas the other studies were conducted in general populations. Data were collected in 2001-2002 (United States), 2007 (Japan), 2010 (France), and 2011 (Netherlands), whereas we collected our data in 2018. Our participants had a higher mean age compared with the studies from United States (42.5 years), Japan (46.4 years), and France (22.6 years) but a lower mean age than the Dutch study (72.0 years).

Nevertheless, our rate might suggest an increase in the impact of internet information on doctor-visiting decisions from 2001 to 2018. The internet has developed substantially and an increasing amount of health information has become available. Furthermore, use of this information has greatly increased, with elderly people as a fast-growing user group [2-5,32,33]. In this perspective, our finding that 25% did change doctor-visiting decisions based on internet information whereas only 10% did so in 2001 supports this trend. It is not possible to judge from this study whether people with diabetes are more or less likely to change their doctor-visiting behavior based on internet information compared with a general population. There is evidence that frequent users of health services, such as people with diabetes or other chronic diseases, are more likely to use the internet for health information compared with nonusers [6,21], which might explain some differences in the rates. On the other hand, a recent study found that demographic differences were more important than the presence or absence of chronic disease in this regard [34]. Other possible explanations might be cultural differences and differences in the level of exposure to internet information, the need for regular as well as irregular doctor visits, and the ability to apply internet information to one’s own health situation.

### Internet-Triggered Changes in Doctor-Visiting Decisions Decreased With Higher Age

Internet-triggered changes in doctor-visiting decisions in either direction decreased significantly with higher age (Table 2). Around 80% of young French adults (mean age 22.6 years) trusted health information from the internet [20], whereas around 40% of older people in the Netherlands (mean age 72.0 years) trusted this information source [27]. Greater trust in information found on the internet among younger people might partly explain our finding, making younger people more able to change doctor-visiting decisions after assessing internet information. Since younger people are more adapted to the internet [35] and the internet might be more tailored for younger users, information might be more easily perceived and transformed according to the individuals’ needs, and thus easier to trust. This point might be reinforced by the lower education in older age groups [36].

### Internet-Triggered Changes in Doctor-Visiting Decisions Increased With the Severity of Anxiety/Depression

In this study, an internet-triggered change in either direction in the decision to visit a doctor increased with the severity of self-reported anxiety/depression, whereas there was no association with self-rated health (Table 2). Others have found a positive relationship between searching the internet for health information and psychological distress [20] and health anxiety [18,37]. As health anxiety levels increase, the relationship between online health information seeking and visiting a doctor based on information found online also increases [38,39]. The internet has the potential to reduce as well as exacerbate health anxiety, and individuals with moderate to high levels of health anxiety experience more anxiety during and after online symptom checking, whereas individuals with low illness anxiety experienced relief [38,40,41]. Naturally, this might lead to changes in doctor-visiting decisions in both directions. Seeking help from the internet for anxiety/depression might partly be explained by the confidentiality of the internet and reduction of stigma. For the same reasons, people with anxiety/depression might avoid doctor visits, in particular if help and support is available online. There are strong indications that anxiety/depression is undertreated [42]; thus, sufficient and adequate treatment should be a concern for health care services and policy makers. Developing a variety of treatment options for people with self-reported anxiety/depression, tailored for individual needs whether online or face to face with a provider, should be a priority.

### Internet-Triggered Decisions Not to Visit a Doctor Were Less Likely Among Men

Men were less likely to change their decision in the direction of not visiting a doctor when they would otherwise have visited one, compared with women. Women still tend to take care of children and other family members’ health more than men do [3,20,35]. Many studies report that women use eHealth more than men do, even if results are not consistent [4]. More searches and findings of adequate health information online among women might be a possible explanation of our result, as women to a larger extent might find what they need for themselves or others’ health issues on the internet and thus decide not to visit a doctor.

### Approximately Half of Participants Never Discussed Internet Information With a Doctor

Approximately half of the study participants (448/858, 52.2%) had never discussed information from the internet with a doctor. Other studies found that 54% in the Netherlands [29], 69% in the United States [23], and 83.5% in Japan [31] had never discussed information obtained from the internet with health professionals. Discussing internet information in the clinical encounter does not seem common, despite some variation. Reported patient-experienced barriers to discussion of internet information include resistance from the physician; disapproval by the physician; fear of embarrassment and of criticizing, offending, insulting, or confronting the physician; and lack of time during the visits [43]. On the other hand, discussions are facilitated by encouragement from the doctor or from online advertisements, by the presence of family members in the consultation, and by higher self-rated ability to appraise internet information and one’s own health status [43]. Some found that men, older people, people with more children under 18 years, and people in poorer health had higher probability of discussing internet information with the doctor [43]. As the internet has developed to be the new “first line” of health services, discussing information from the internet with a doctor (the next level) might not be necessary as long as internet information is considered understandable and sufficient and contributes in solving the patients’ problem without a doctor’s visit.

### Higher Probability of Discussing Internet Information With a Doctor for People With Bad/Very Bad Health and Moderate Anxiety/Depression

The probability of discussing internet information with a doctor increased for those in bad/very bad self-rated health (compared with excellent health) and for those with moderate self-reported anxiety/depression (compared with no anxiety/depression). Discussing with a doctor presupposes doctor visits. People with bad/very bad self-rated health and moderate anxiety/depression are likely to meet this requirement, as doctor visit rates might be higher in these groups. We did not find an association between discussing internet information with a doctor for those with severe anxiety/depression, possibly due to a heavy disease burden that might hinder doctor visits [42,44] and probably decrease internet searching as well.

### Implications and Further Research

Understanding patterns of health care–seeking behavior in the digital era is important for planning of health services for the population. Our study indicates that doctors should be aware of the need to discuss information from patients’ internet use in the clinical encounter. Also, more effort should be put in providing high-quality updated online information for patients. Such information might give good advice regarding the need for doctor visits and thus lead to more tailored use of health care services. This might be particularly important for patients with anxiety/depression.

This study did not address any possible associations between changing doctor-seeking behavior and discussing internet findings with a doctor, which would be interesting for future research. Did those who decided to visit a doctor based on information from the internet discuss the information that changed their decision? Future research should also include investigations of specific webpages used for health information, patient rating of the information quality, associations with the use of health care services, and other impacts of internet use like, for instance, lifestyle changes.

### Limitations and Strengths

This study had some shortcomings, which were explored in detail in our first publication in the DIAcare project [6]. Summing up, we discussed the low estimated participation rate (the main limitation); distribution of the questionnaire by email; recall bias; the validity of self-reported data; the cross-sectional study design; and participation related to gender, age, health, socioeconomic group, and interest in the subject studied. Our conclusion was that younger individuals might be overrepresented but that it is not possible to judge the magnitude or direction of a possible nonresponse bias because different factors might pull the tendency in different directions or level each other out. The low response rate is in itself not an indication of low representativeness, as nonresponse bias may be a problem even if response rates are high. We suggested that nonresponse bias posed a limited threat to our study’s validity; however, generalization should be made with caution.

Worries, anxiety/depression, and emotional distress are not defined according to diagnostic manuals in this study and rely solely on self-report. Since many people with different kinds of psychological distress do not seek help and thus are undiagnosed [42], we consider self-report to be interesting as such in this field and do not think that this has distorted the validity of our results.

It should also be mentioned that this study does not address whether participants used the internet frequently or infrequently, which could have contributed to explaining the results. Likewise, we cannot exclude other unmeasured confounders of the reported associations, such as accessibility of doctor visits and doctor characteristics.

This study also has some strengths, which are similar to the strengths discussed in the first paper in this project [6]. The most important strength is the focus on a scarcely investigated research field. Other strengths are the detailed questionnaire specifically tailored to people with diabetes, the recruitment of participants from all of Norway, the inclusion of a wide age span of participants, and that we were able to analyze the data shortly after they were collected. Finally, the cooperation with NDA made real user participation possible in the design, execution, and implementation of the study.

### Conclusions

Our findings suggest that the use of eHealth seems to have a significant impact on doctor- visiting decisions among people with diabetes, especially among younger people (aged 18 to 39 years) and those reporting anxiety/depression. It is of great importance that the information posted is of high quality and that the large differences between eHealth users regarding age as well as mental and somatic health status are taken into account. Furthermore, we conclude that around half of the participants did not discuss information from the internet with a doctor. More research is needed to confirm and further explore the findings of this study.

## Appendix

Multimedia Appendix 1 Questionnaire.

## Abbreviations

GP: general practititioner
NDA: Norwegian Diabetes Association
NSD: Norwegian Centre for Research Data
OR: odds ratio
T1D: type 1 diabetes
T2D: type 2 diabetes
